# Penile length of newborns and children in Surakarta, Indonesia

**DOI:** 10.1186/1687-9856-2013-S1-P187

**Published:** 2013-10-03

**Authors:** Annang Giri Moelyo, Melita Widyastuti

**Affiliations:** 1 Sebelas Maret University Moewardi Hospital, Surakarta, Indonesia

**Keywords:** penile length, newborns, children

## 

Normal penile length reference is required for assessing a child with micropenis complaint. Some studies revealed differences in normal penile length based on their race. We want to know the normal penile length of newborns, children and adolescent in Surakarta which majority people are Javanese.

We studied male newborns and children attended to Moewardi Hospital in the period of January 2011 – January 2012. We excluded chronic diseases, hypospadia, ambiguous genitalia, any congenital anomalies and syndromes (such as Down syndrome). We stretched flaccid penile lengths, measured with wooden spatulas, depressed the pubic fat and placed vertically along the dorsal penis. The penile length was measured from basis of penis until gland without preputium. It was done three times. The data were analyzed by SPSS 17.0 version.

There are 300 subjects, 100 newborns and 200 children. Two hundreds and ninety six subjects (98,7%) are Javanese. The mean of penile length of preterm (30-36 weeks) and aterm (>36 weeks) newborns are 1.88 ± 0.14 and 2.37 ± 0.26 cm, respectively. The mean of penile length of 0-<6 months; 6-<12 months, 1-<3 years; 3-<5 years; 5-<7 years; 7-<9 years; 9-<11 years; 11-<13 years; 13-<15 years and 13-18 years are 2.67+0.58; 2.67+0.58; 2.80+0.84; 3.5+0.55; 3.5+0.71; 3.85+0.53; 4.50+0.71; 4.63+1.13; 5.53+1.45; and 6.16+1.19 cm. Our study revealed that normal penile length in Javanese children are smaller than normal range reference we used from Moore WT and Eastman RC.

